# The Role of Heparanase in Pulmonary Cell Recruitment in Response to an Allergic but Not Non-Allergic Stimulus

**DOI:** 10.1371/journal.pone.0127032

**Published:** 2015-06-03

**Authors:** Abigail Morris, Bo Wang, Ida Waern, Radhakrishnan Venkatasamy, Clive Page, Eric P. Schmidt, Sara Wernersson, Jin-Ping Li, Domenico Spina

**Affiliations:** 1 Sackler Institute of Pulmonary Pharmacology, Institute of Pharmaceutical Science, King’s College London, London, United Kingdom; 2 Department of Medical Biochemistry and Microbiology, Uppsala University, Box 582, Uppsala, Sweden; 3 Department of Anatomy, Physiology and Biochemistry, Swedish University of Agricultural Sciences, Box 7011, Uppsala, Sweden; 4 Program in Translational Lung Research, University of Colorado School of Medicine, Aurora, CO, United States of America; French National Centre for Scientific Research, FRANCE

## Abstract

Heparanase is an endo-β-glucuronidase that specifically cleaves heparan sulfate proteoglycans in the extracellular matrix. Expression of this enzyme is increased in several pathological conditions including inflammation. We have investigated the role of heparanase in pulmonary inflammation in the context of allergic and non-allergic pulmonary cell recruitment using heparanase knockout (Hpa^-/-^) mice as a model. Following local delivery of LPS or zymosan, no significant difference was found in the recruitment of neutrophils to the lung between Hpa^-/-^ and wild type (WT) control. Similarly neutrophil recruitment was not inhibited in WT mice treated with a heparanase inhibitor. However, in allergic inflammatory models, Hpa^-/-^ mice displayed a significantly reduced eosinophil (but not neutrophil) recruitment to the airways and this was also associated with a reduction in allergen-induced bronchial hyperresponsiveness, indicating that heparanase expression is associated with allergic reactions. This was further demonstrated by pharmacological treatment with a heparanase inhibitor in the WT allergic mice. Examination of lung specimens from patients with different severity of chronic obstructive pulmonary disease (COPD) found increased heparanase expression. Thus, it is established that heparanase contributes to allergen-induced eosinophil recruitment to the lung and could provide a novel therapeutic target for the development of anti-inflammatory drugs for the treatment of asthma and other allergic diseases.

## Introduction

Heparan sulfate proteoglycans consist of a protein core decorated with heparan sulfate linear polysaccharides. This family includes syndecans, glypicans and secreted forms of extracellular matrix proteoglycans (e.g. perlecan). Heparan sulfate can bind to a range of proteins (e.g. chemokines, growth factors, growth factor receptors) and thereby fine tune signaling within the extracellular matrix [1,2]. Manipulating the expression pattern of these proteoglycans highlights the critical role of these macromolecules in growth and development, tissue injury and repair, cell signaling, extracellular matrix barrier function and tumor metastasis. For example, mice deficient in heparan sulfate proteoglycan are embryonically lethal [3], whilst mice deficient in perlecan have impaired angiogenesis and reduced wound healing [4]; syndecan-1 is also implicated in wound healing and angiogenesis [5,6]. However, few studies have examined the role of heparan sulfate proteoglycans in pulmonary inflammation. Mice deficient in syndecan-1 were associated with an increase in eosinophil recruitment to the airways and bronchial hyperresponsiveness, whilst soluble forms of syndecan-1 administered to allergic mice were protective [7]. Syndecan-1 also binds to neutrophil elastase and promotes an inflammatory milieu [8].

Mammalian heparanase is an enzyme that specifically cleaves heparan sulfate chains. This enzyme was first cloned in 1999 and is the only known mammalian enzyme capable of disassembling heparan sulfate [9,10] and is expressed in many inflammatory cells including dendritic cells, T and B cells, platelets, macrophages, neutrophils and mast cells and is implicated in mediating diapedesis and recruitment of leukocytes to inflammatory sites [11]. Increased expression of heparanase in various tumours correlates with tumour invasiveness, angiogenesis and poor prospective survival. There is also evidence of increased heparanase expression in inflammatory bowel disease [12] and in the synovium of rheumatoid arthritis patients [13]. Heparanase overexpression in mice led to an exacerbation of delayed type hypersensitivity (DTH) reaction in the skin [14] and may specifically regulate transmigration of monocytes in this inflammatory response [15]. Few studies have examined the role of this enzyme in regulating pulmonary recruitment of inflammatory cells. This is relevant to an understanding of the pathophysiology of inflammatory diseases like asthma and COPD which are characterized by the presence of inflammatory cells like eosinophils and neutrophils. It has been reported that mice deficient in heparanase are protected from sepsis-induced acute lung injury [16] and allergic inflammation [17], suggesting that heparan sulfate provides a pro-inflammatory cue. However, elimination of syndecan-1 appears to lead to an exacerbation of the allergic inflammatory response [7]. Hence, we hypothesize that heparanase could play a role in inflammatory cell migration in lung inflammation and used heparanase knockout (Hpa^-/-^) mice and pharmacologically treated mice as two models to investigate the role of heparanase in pulmonary inflammation and changes in pulmonary lung mechanics.

## Methods

### Human lung tissue

Lung tissue from human subjects (anonymized) was obtained from the National Jewish Health, Denver USA (normal controls) and from the Lung Tissue Repository Consortium (COPD lungs) [18] and tissue heparanase expression was detected using a previously described method [19].

Lung samples were obtained either from surgical lung biopsy or explants at the time of lung transplantation. Severity of COPD was stratified according to the 2013 Global Initiative for Obstructive Lung Disease (www.goldcopd.com) guidelines, which ranged from Stage 0 (normal spirometry with chronic symptoms), Stage I (FEV1/FVC < 70% with FEV1 > 80% predicted), Stage II (FEV1/FVC < 70% with FEV1 50–79% predicted), Stage III (FEV1/FVC < 70% with FEV1 30–69% predicted), and Stage IV (FEV1/FVC < 70% with FEV1 < 30% predicted). Samples were de-identified, and banked samples obtained via, and approved by, the Colorado Multiple Institutions Review Board (number 08–1462). We performed immunofluorescence on 4-μm paraffin-embedded sections using a rabbit polyclonal antibody to human heparanase (Prospec, Ins-26-2, 1:1000). Rabbit IgG (Abcam, 27472, 1:1000) served as an isotype control. We measured intensity of staining using Metamorph (Molecular Devices, Sunnyvale, CA), as previously described [19].

### Animal Welfare and Maintenance

Female BALB/c mice (18-20g; Charles River or Harlan, UK) were housed in rooms maintained at a constant temperature (21 ± 2°C) and humidity (55 ± 15%) with a 12 hour light-dark cycle. Animals had food (SDS, UK) and water available *ad libitum*. All experiments were performed under The Scientific Procedures (Animals) Act 1986 and a Home Office project licence (PPL 70/8021) approved by the Animal Welfare and Ethical Review Board at King’s College London. Studies using mice deficient in heparanase [20] were carried out at the Department of Medical Biochemistry and Microbiology, Uppsala University, Uppsala, Sweden and approved by Uppsala Ethical Committee on Animal Experiments (ethical approval number; C228/12 and C135/8). Animals had free access to food and water and were housed with suitable enrichment and bedding with 12h day/night cycle. All surgery was performed under general anaesthesia (King’s College London, urethane; Uppsala University, pentobarbital sodium) and all efforts made to minimize suffering.

### Induction of non-allergic airway inflammation

In order to establish lung inflammation, mice were anaesthetised with isoflurane and dosed intranasally (i.n.) with either zymosan (4 mg/kg) (Sigma-Aldrich, UK) or LPS (1.25 mg/kg) (from Escherichia Coli, Sigma-Aldrich, UK). Control mice received the same volume (i.n) of sterile saline.

### Induction of allergic airway inflammation

**Fig 1:**
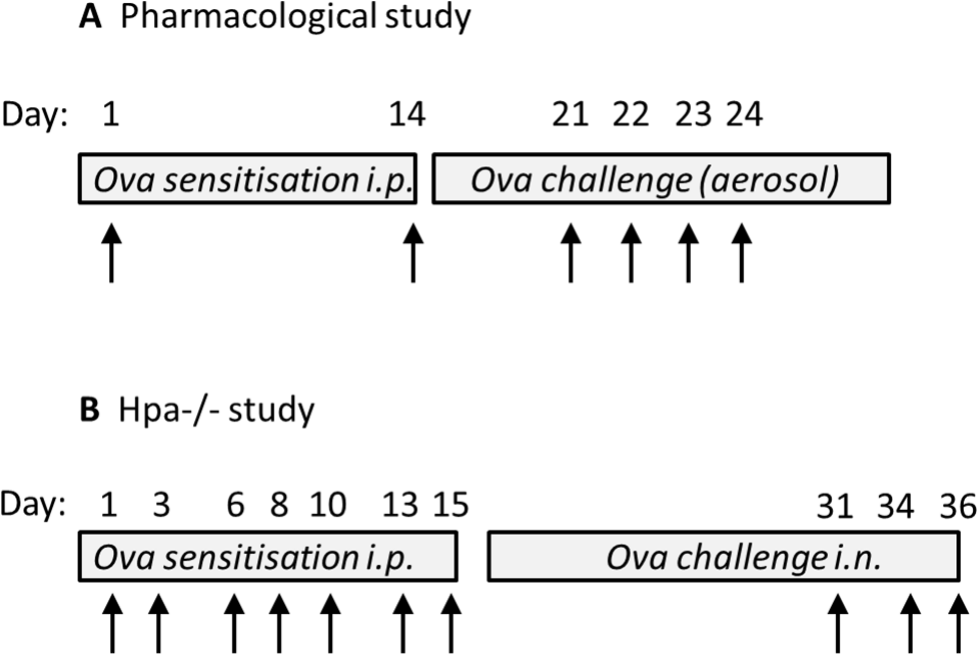
Protocol for allergen sensitization and challenge. Diagrammatic representation of the sensitization and challenge protocols to induce allergic lung inflammation in WT BALBc (A) and Hpa^-/-^ (B) mice. Arrow represents exposure to ovalbumin (OVA). Biological readouts were undertaken 24 h following the last OVA administration. In (A), bronchoalveolar lavage and lung function was measured on day 24 and day 25 respectively. In (B), BAL and serum samples were collected on day 37.

For pharmacological studies, allergic airway inflammation was induced in WT BALB/c mice. In these studies, mice (6–8 weeks) were sensitised with ovalbumin (OVA, i.p.) (Chicken ovalbumin, grade V. Sigma, UK). Each mouse received 30 μg of OVA absorbed in 16 μL of aluminium hydroxide (Ems, Brazil) in saline to give a final volume of 0.4 mL. Sham mice received saline and aluminum hydroxide alone. Control and OVA mice were sensitised on day 1 and boosted on days 7 and 14. On days 21–23 (BAL) or 21–24 (lung function), OVA-sensitised mice were exposed to 3% OVA by aerosol in a small chamber attached to a nebuliser (DeVillbisS 99; HCE, UK). On each challenge day mice were exposed to OVA for 30 minutes (Fig 1A).

In a separate series of experiments, allergic airway inflammation was induced in Hpa^-/-^ mice backcrossed for more than 10 generations to the C57BL/6J genetic background [20]. Experimental groups were age and sex matched (WT control: 6 **♀,** 5 **♂**; WT OVA: 7 **♀,** 5 **♂**; Hpa^-/-^ control: 5 **♀,** 2 **♂**: Hpa^-/-^ OVA: 5 **♀,** 4 **♂**) and WT littermates were used as controls. In these experiments, mice (10–14 weeks) were immunized i.p. with 10 μg of OVA (Sigma-Aldrich) in 100 μl of PBS on days 1, 3, 6, 8, 10, 13 and 15. Control mice were non-immunized. On days 31, 34 and 36, mice were anaesthetized with isofluorane (4%) for intranasal administration with 20 μg of OVA in 20 μL of PBS (Fig 1B).

### Pharmacological intervention

Mice were subcutaneously (s.c.) administered with the heparanase inhibitor (Muparfostat, PI-88; 30 – 100 mg/kg: Progen Pharmaceuticals, Brisbane Australia) or vehicle either once (q.i.d.) or twice (b.i.d.) a day in the non-allergic model or daily in the case of the allergic inflammatory model. In the case of allergic studies, animals were treated on 3 (eosinophil recruitment) or 4 (lung function) consecutive days.

### Assessment of Lung Function

Animals were anaesthetised with urethane (2 g/kg) i.p. 24 hours after the last OVA challenge and lung function was measured using a previously described technique [21]. A tracheotomy was performed and a cannula inserted and sutured in place. Mice were then placed in a plethysmograph chamber via the cannula connected to a 4-way manifold with one port attached to a differential pressure transducer (± 20 cm H2O: Validyne, UK). Two ports were connected to the inspiratory and expiratory ports of a volume cycled ventilator (CWA Incorporated, USA). Mice were ventilated at 150 breaths/minute with a tidal volume of 0.15–0.20 mL and a positive expiratory pressure between 3 and 5 cm H_2_O. Transpulmonary pressure was estimated as the difference between mouth pressure and box pressure, as the chest wall contributes little to the overall compliance of the respiratory system. Changes in flow were determined with a Fleisch pneumotachograph connected to a side port of the chamber. Flow was measured with a differential pressure transducer (± 20 cm H_2_O: Validyne, UK). The flow was recorded to give a continuous reading of tidal volume. Breath-by-breath recording of total lung resistance (RL: cmH_2_0.s/L) was calculated by an online respiratory analyser on a computer (LFR 7; Mumed Ltd., UK).

Airway responsiveness was assessed by inhaled aerosolised methacholine at a concentration range of 3.125–50 mg/mL. Aerosols of methacholine generated from an ultrasonic nebulizer were administered directly to the lungs via the cannula and a port of the plethysmograph. Aerosolised solutions were delivered to the mouse for 8 seconds, at a rate of 150 breaths/minute with a tidal volume of 0.15–0.2 mL. Total airway resistance was measured prior to, and following administration of saline and then following administration of methacholine. Upon completion of experiments, animals were humanely killed by cervical dislocation. The provocative concentration (PC) of methacholine which caused a 100% increase in post saline RL (RL PC100) and peak response (i.e. maximum percentage change in post saline RL) for each animal was used as a measure of airway sensitivity and reactivity, respectively.

### BAL fluid analysis

Mice were humanely killed 24 h after receiving the inflammatory stimulus with an intraperitoneal (i.p) injection of 0.2 mL 50% urethane (King’s College London) or 40 – 60 mg/kg pentobarbital sodium (Uppsala University). Lungs were lavaged via a cannula inserted into the trachea and instilled with 3 x 0.5 mL of sterile saline (King’s College London) or with 2 x 1 mL of sterile HBSS (Uppsala University). In experiments using Hpa^-/-^ allergic mice, BAL fluid analysis was performed as previously described [22]. For differential cell counts, cytospin slides were prepared using 150 μL of BAL fluid spun at 1000 x g for 1 minute. Cytospins were allowed to dry before staining using a Diff-Quick stain. Differential cell counts were performed according to standard morphological criteria on Diff-Quick stained cytospins (100 cells/sample). Cells were identified as mononuclear cells, neutrophils and eosinophils. Almost all mononuclear cells observed were macrophages, but for accuracy and clarity of presentation, were analyzed and analysed as mononuclear cells. To perform total cell counts, 50 μL of BAL fluid was mixed well with 50 μL of T**ü**rk solution (Merck, Germany) and counts performed using a haemocytometer. The total number of cells counted was then standardised to the number of total cells x 10^6^/mL. In both instances, the individual counting the cells was blind to treatment or genotype.

### IgE ELISA

IgE anti-OVA ELISA were performed as previously described in allergic WT and Hpa^-/-^ mice [22]. For analysis of OVA-specific IgE in sera, 96-well microtiter plates (Nunc) were incubated overnight at 4°C with goat anti-mouse IgE coating antibody (10 μg/mL, 100 μL) from the Mouse IgE ELISA Quantification Kit (Bethyl Laboratories). Plates were washed three times with 200 μL washing buffer (50 mM Tris, 0.14 M NaCl, 0.05% Tween-20, pH 8) and then incubated with 200 μL blocking buffer (50 mM Tris, 0.14 M NaCl, 1% BSA, pH 8) for 2 h in room temperature. Mouse sera were diluted 1:3 in blocking buffer and incubated for 2 h (100 μL). Plates were washed and bound anti-OVA antibodies were quantified by first incubating with biotinylated OVA (2 μg/mL, 100 μL) for 60 minutes. Plates were then washed five times before adding strepavidin-peroxidase conjugate (0.05 U, 100 μL, Roche Diagnostics) and incubating for 30 minutes. After five washes, 3,3′,5,5’-tetramethylbenzidine liquid substrate (100 μL, Sigma-Aldrich) was added. The reaction was stopped after 30–45 minutes by adding 100 μl 2M H_2_SO_4_ and plates were read at 450 nm.

In the biotinylation procedure 10 mg OVA (Grade V, Sigma-Aldrich) in 1 mL PBS was mixed with 2.5 mg biotinamidocaproic acid 3-sulpho-N-hydroxy-succinimide ester (Sigma-Aldrich) dissolved in 0.25 mL distilled water. The mixture was stirred for 2 h at room temperature. To remove unreacted biotin the mixture was dialysed against PBS at 4°C and then the biotinylated OVA was stored at 4°C in 0.1% sodium azide.

### Statistical Analysis

Data was expressed as median, with interquartile range unless otherwise specified. Cell data was analyzed using non-parametric methods (Kruskal Wallis, and Mann Whitney U test). In some instances, neutrophil or eosinophil recruitment to the lung was expressed as a percentage in terms of the average number of total cells. Analysis of variance with Dunnett’s post hoc test was used to analyze RL PC100 (log transformed) and peak response in RL. Data was analyzed using GraphPad Prism version 5. P values of < 0.05 were considered to be significant.

## Results

### Non-allergic inflammation in Hpa^-/-^ mice

#### Zymosan induced inflammation

**Fig 2:**
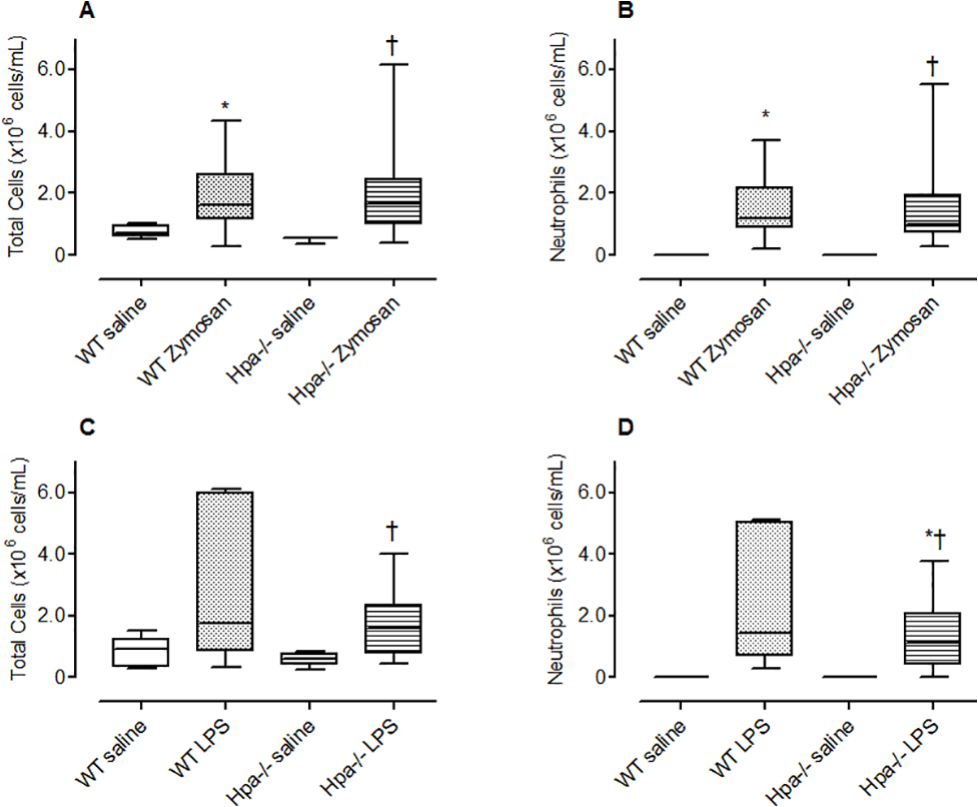
The effect of genetic modification of heparanase on neutrophil recruitment to the lung. The total number of inflammatory cells (A, C) and neutrophils (B, D) recruited to the lung following acute exposure of wild-type (WT), and Hpa^-/-^ mice to intranasal (i.n.) saline, zymosan (A, B) or LPS (C, D). Data presented as box plots (median, 25–75 percentile) with whiskers representing 5–95% confidence interval. The number of animals represented by box plots in each panel is as follows; Panel A, B: Saline treated group (WT; n = nil ♀, 5 ♂; Hpa^-/-^, n = 2 ♀, 1 ♂) and zymosan treated group (WT; n = 4 ♀, 4 ♂; Hpa^-/-^, n = 3 ♀, 4 ♂). *p < 0.05 compared with saline; †No significant difference compared with WT zymosan. Panel C, D: Saline treated group (WT: wild type; n = 2 ♀, 3 ♂; Hpa^-/-^, n = 3 ♀) and LPS treated group (WT; n = 5 ♀; Hpa^-/-^, n = 4 ♀). *p < 0.05 compared with saline; †No significant difference compared with WT LPS. Data obtained from one experiment.

The total number of cells in the BAL fluid of saline injected WT mice (median, 25–75 percentile; 0.72 (0.60 – 0.96) x 10^6^ cells/ml, n = 5) was predominantly comprised of mononuclear cells and represented 99% of the total cell population with an absence of neutrophils. There was a significant increase in total number of cells (Fig 2A, p = 0.038) and neutrophils (Fig 2B, p = 0.0016) recruited to the airways following challenge with zymosan. There was no significant difference in the number of total cells (Fig 2A) or neutrophils (Fig 2B) between Hpa^-/-^ mice and WT mice indicating that heparanase is not involved in the recruitment of inflammatory cells to the lung following an acute challenge with zymosan.

#### Lipopolysaccharide-induced lung inflammation

The total number of cells in the BAL fluid of saline injected WT mice (median, 25–75 percentile: 0.92 (0.35 – 1.26) x 10^6^ cells/ml, n = 5) was predominantly comprised of mononuclear cells and represented 99% of the total cell population with an absence of neutrophils. There was a tendency (although not statistically significant) increase in the total number of cells (Fig 2C, p = 0.0652) and a significant increase in neutrophils (Fig 2D, p = 0.0005) recruited to the airways following challenge with LPS. There was no significant difference in the number of total cells (Fig 2C) or neutrophils (Fig 2D) between Hpa^-/-^ mice and WT mice indicating that heparanase is not involved in the recruitment of inflammatory cells to the lung following an acute challenge with LPS.

#### The effect of Muparfostat on non-allergic lung inflammation

**Fig 3:**
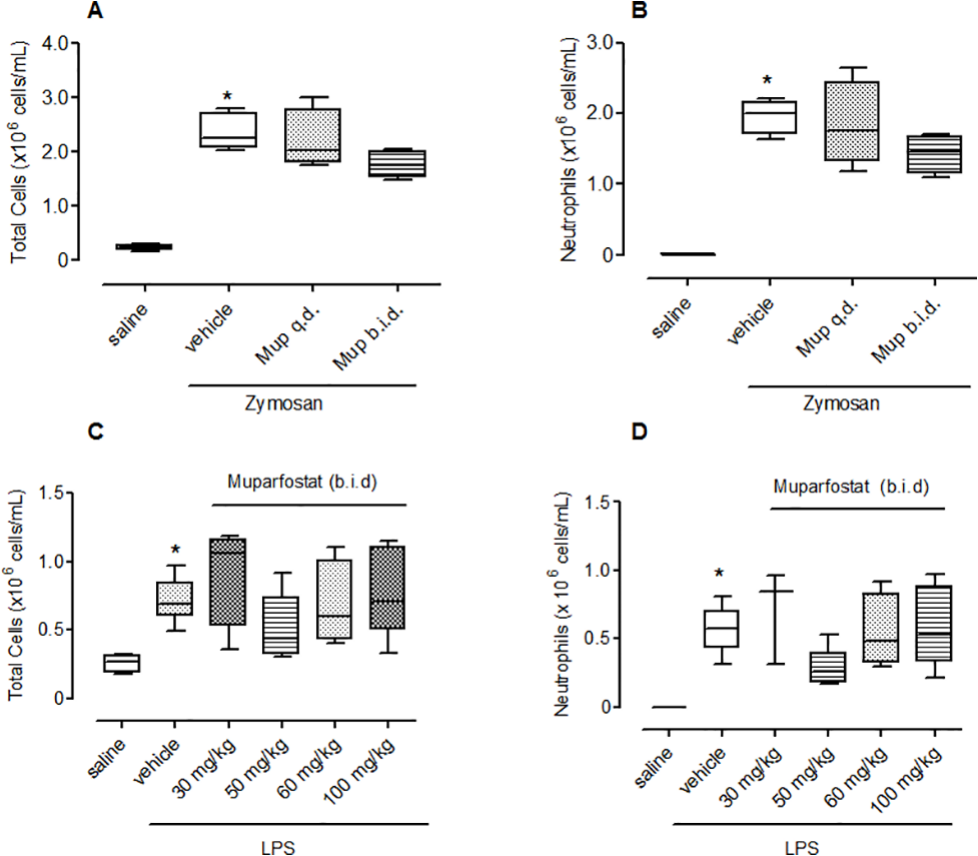
The effect of pharmacological treatment with the heparanase inhibitor Muparfostat on neutrophil recruitment to the lung. The total number of inflammatory cells (A, C) and neutrophils (B, D) recruited to the lung following acute exposure to zymosan (A, B) or LPS (C, D). Wild-type (WT), and Hpa^**-/-**^ mice received intranasal (i.n.) saline, zymosan (A, B) or LPS (C, D). The number of total cells (A) and neutrophils (B) in bronchoalveolar lavage fluid 24 h after intranasal administration of zymosan in BALB/c mice treated with Muparfostat (30 mg/kg s.c.) dosed 30 minutes before (q.d.) and 6 h after (b.i.d) zymosan exposure, n = 4 per group. Data presented as box plots (median, 25–75 percentile) with whiskers representing 5–95% confidence interval. *p < 0.05 compared with saline. Data obtained from one experiment. In other experiments total number of cells (C) and neutrophil (D) recruitment to the lung was measured following intranasal administration of LPS in vehicle and Muparfostat (b.i.d.) treated mice (n = 3–9). Data presented as box plots (median, 25–75 percentile) with whiskers representing 5–95% confidence interval. *p < 0.05 compared with saline. Treatment groups were not significantly different from vehicle (symbol not reported). Data was obtained from two independent experiments.

The majority of the cells in the saline treated animals were predominantly comprised of mononuclear cells (> 95%), but in zymosan vehicle treated animals neutrophils comprised 85% of the total cell number. Treatment of mice with the heparanase inhibitor muparfostat once a day (q.d) or twice a day (b.i.d) did not significantly alter the total number of inflammatory cells (Fig 3A) or neutrophils (Fig 3B) infiltrating the lung in response to zymosan.

Similarly, intranasal administration of LPS also caused a significant increase in total cell recruitment to the lung due to the infiltration of neutrophils (80% of total cell count). Muparfostat did not significantly reduce total number of cells (Fig 3C) or neutrophils (Fig 3D) in response to LPS.

### Heparanase expression in human lung tissue

**Fig 4:**
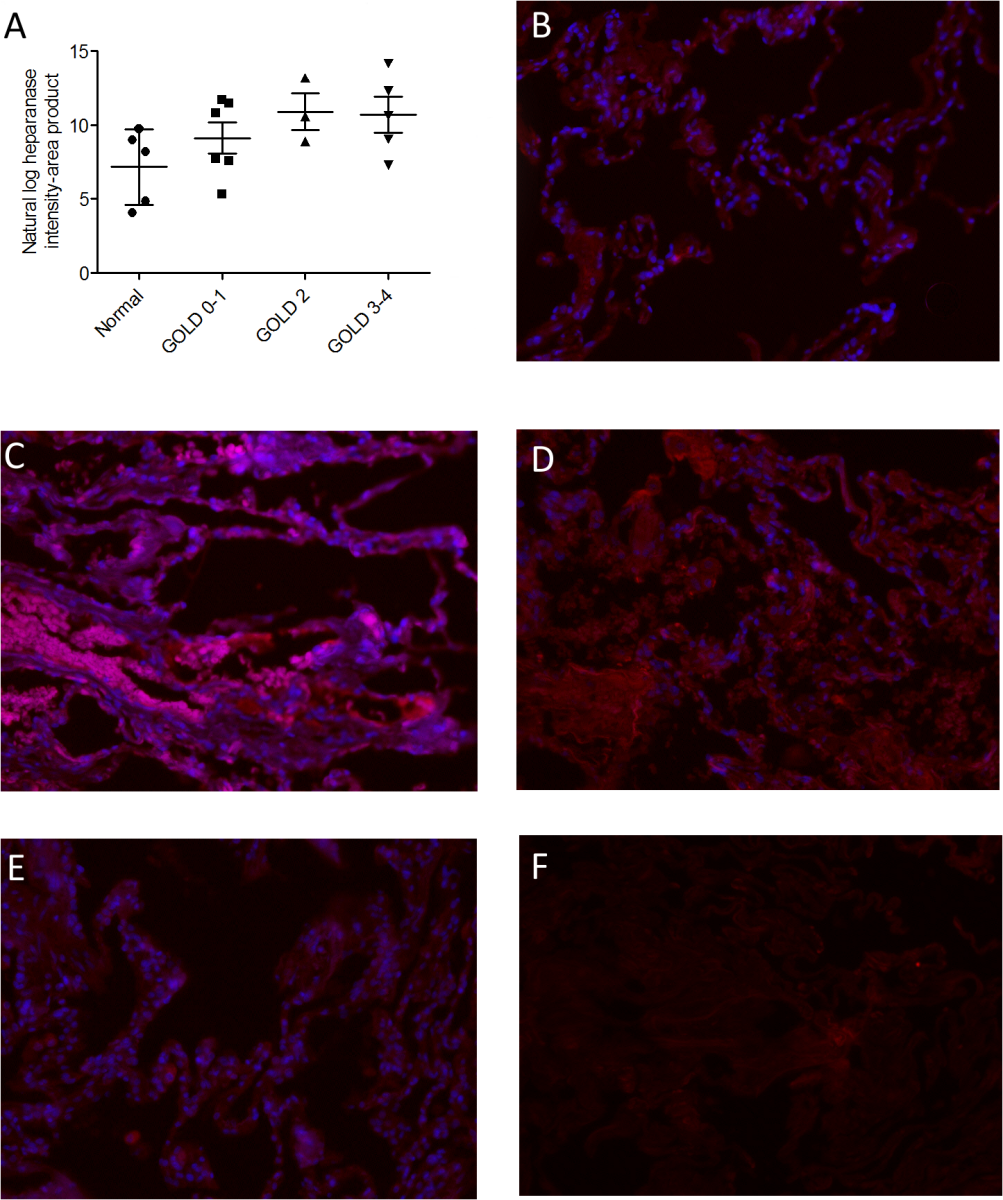
Heparanase expression in lung tissue from normal subjects and COPD patients of varying severity. Each point represents the intensity of staining for heparanase expression in normal human lung tissue and in subjects with varying severities of COPD (A). Representative images are shown (B-F reflecting Gold 0–1, Gold 2, Gold 3–4, healthy subject, and isotype control, respectively. Red: heparanase; blue: DAPI (DAPI not performed for isotype control). Image brightness was uniformly adjusted for clarity and all heparanase quantification was performed using unadjusted (raw) images. Magnification was 40 x for all images. The data was analyzed as continuous variables and there was a significant linear trend (post-ANOVA, r-squared = 0.27 and p = 0.029). Horizontal line represents mean and limits represent SEM.

Available clinical data from de-identified healthy and COPD cohorts providing lung tissue are shown in Table 1. Immunofluorescence revealed that heparanase expression increased with sequential increases in GOLD stage (Fig 4, linear trend by ANOVA p = 0.029).

**Table 1:** Patient demographics of COPD patients.

Group	Gender (F/M)	Age (mean, (SD))	% FEV1 (pre)	% FEV1 (post)	Pack years
**GOLD (0–1)**	2/4	56 (8)	89 (78–100)	94 (85–100)	39 (4–74)
**GOLD (2)**	1/3	51 (6)	48 (38 – 59)	54 (46–63)	30 (-3 – 63)
**GOLD (3–4)**	2/3	54 (4)	24 (8 – 41)	46	48 (15–81)

% predicted pre and post bronchodilator

Spirometry data not available for healthy donors

### Heparanase plays a role in allergic inflammation

**Fig 5:**
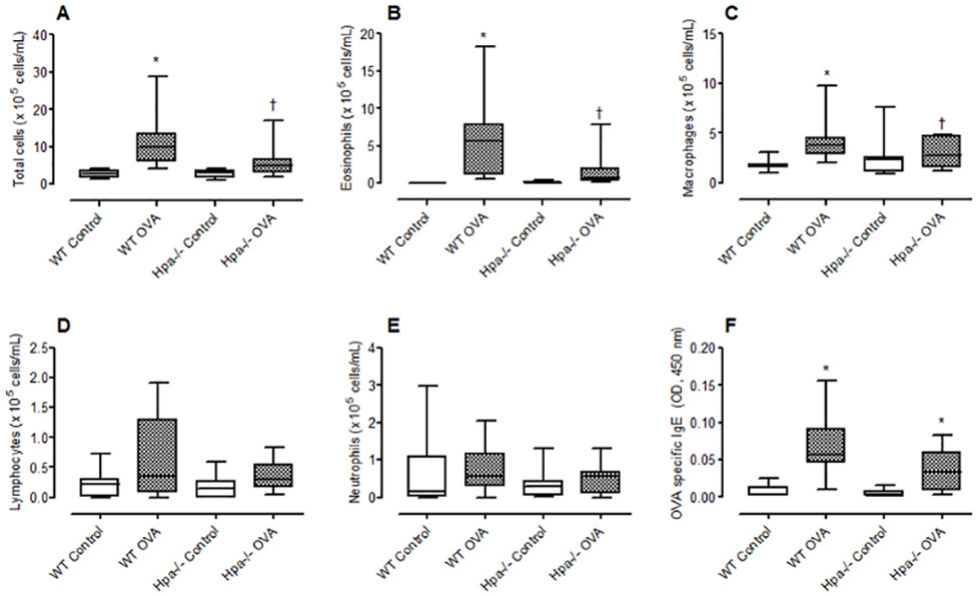
Effect of heparanase gene deletion on allergic inflammation induced by ovalbumin (OVA). Box plots showing (A) total cell, (B) eosinophil, (C) macrophages, (D) lymphocytes, (E) neutrophils in bronchoalveolar lavage fluid and (F) OVA specific IgE in serum in control and OVA immunized and challenged mice. Mice were sensitized by repeated i.p. injections with 10 μg OVA or were non-sensitized (controls). All mice received three i.n. instillations with 20 μg OVA and lavage was undertaken 24 h after the last intranasal administration of OVA. Data expressed as blox plots (median, 25–75 percentile) with whiskers representing 5–95% confidence interval (7–12 animals for cell data and 6–10 for IgE data). *p < 0.05 compared with control; †No significant difference compared with Hpa^-/-^ control. Data pooled from four independent experiments.

Adult WT mice immunized with OVA and repeatedly exposed to this antigen induced an allergic inflammatory response (Fig 5) characterized by an eosinophilic rich cell infiltrate (42% of total cells, n = 12). The significant increase in total cells (Fig 5A, p < 0.05), eosinophils (Fig 5B, p < 0.05) and macrophages (Fig 5C, p < 0.05) following OVA challenge in wild type mice was inhibited in Hpa^-/-^ mice. Neutrophil and lymphocyte recruitment to the airways were not significantly increased following antigen challenge, and the absence of heparanase did not affect the migration of these cell types into the airways. Th2 cell activation was indirectly assessed by measurement of OVA specific IgE, which was significantly elevated in WT and Hpa^-/-^ OVA sensitized and challenged mice compared with their respective controls (Fig 5).

#### The effect of Muparfostat against OVA induced allergic lung inflammation

**Fig 6:**
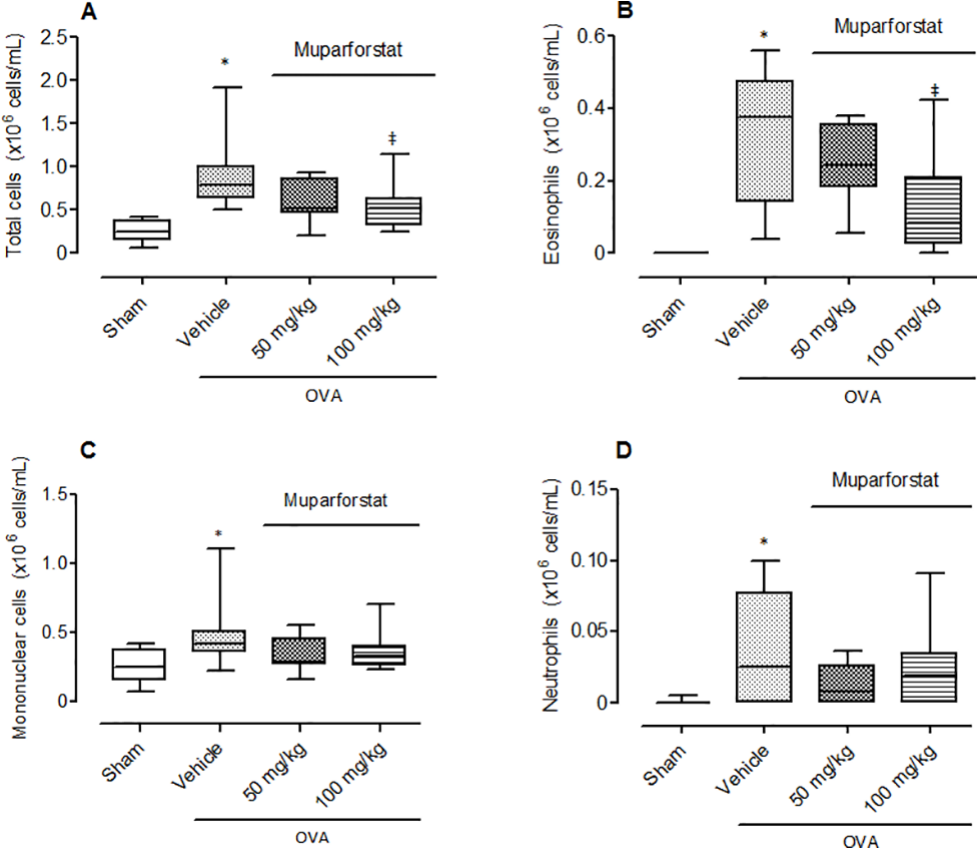
Effect of Muparfostat on allergic inflammation induced by OVA. Box plots showing (A) total cell, (B) eosinophil, (C) mononuclear cell and (D) neutrophil cell number in the lungs of mice following sensitisation and aerosol challenge with OVA. Mice were treated with Muparfostat (50 mg/kg and 100 mg/kg s.c.) 30 minutes before challenge. Mice were challenged with aerosolised 3% OVA for three consecutive days and lavage undertaken 24 h after the last challenge. Data expressed as blox plots with whiskers representing 5–95% confidence interval (n = 9–15). *p < 0.05 compared with sham and ‡p < 0.05 compared with vehicle. Data obtained from four independent experiments.

Mice treated with OVA and vehicle showed a significant increase in the total number of cells compared to sham animals (Fig 6A, p < 0.05). Eosinophils comprised 36% of the total cell infiltrate (Fig 6B, p < 0.05). In addition, there was also a small and significant increase in the number of neutrophils and mononuclear cells in the OVA vehicle treated group compared to sham (Fig 6C and 6D). The heparanase inhibitor Muparfostat at the highest dose evaluated caused a significant inhibition of eosinophil recruitment to the airways (Fig 6B).

#### The effect of Muparfostat against OVA induced allergen-induced bronchial hyperresponsiveness

**Fig 7:**
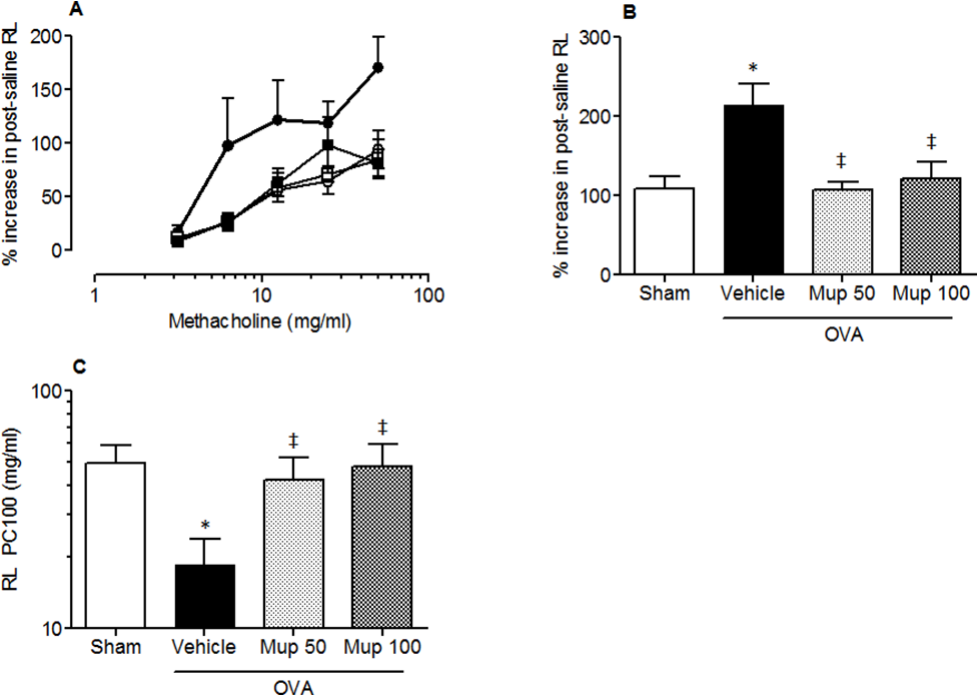
Effect of Muparfostat on OVA induced changes in lung function. Graphs showing (A) % increase in total lung resistance versus methacholine dose in sham immunized (○), OVA sensitized in vehicle-treated (●) or OVA-immunized mice treated with Muparfostat 50 mg/kg (Mup, □) or Muparfostat 100 mg/kg (Mup, ■) s.c. before being challenged with aerosolised 3% OVA for 4 consecutive days, (B) peak percentage increase in post saline resistance, (C) R_L_ provocative concentration (PC)100 for methacholine. Each point or columns represent mean ± SEM n = 8–11, p < 0.05 versus sham* or vehicle‡. Data obtained from four independent experiments.

Methacholine caused a dose-dependent increase in post-saline resistance (RL) (Fig 7A). The percentage increase in post-saline resistance (%R_L_) of the lungs (i.e. peak response) to increasing concentrations of methacholine was significantly greater in OVA vehicle treated animals compared with sham sensitised animals (mean ± s.e.m; 107.6 ± 16.8, n = 10 versus 213.8 ± 26.4, respectively, n = 11 p < 0.05; Fig 7B). In animals treated with muparfostat 50 mg/kg and 100 mg/kg there was a significant decrease in the peak response (107 ± 10.1, n = 8, p < 0.01 and 121 ± 21.7, n = 11 p < 0.05 respectively for dose). Similarly, airway sensitivity to methacholine (RL PC100, mg/mL) was reduced by approximately 3 fold in OVA vehicle treated animals compared with sham sensitised animals (mean ± s.e.m; 18.5 ± 5.2, n = 10 versus 49.7 ± 9.5 respectively, n = 10, p < 0.05; Fig 7C) indicating an increase in airway sensitivity to methacholine. This increase in airway sensitivity was significantly inhibited in OVA sensitized and challenged mice, treated with Muparfostat (50 mg/kg or 100 mg/kg; Fig 7C). The R_L_ PC100 (mg/mL) was significantly increased compared with vehicle treated sensitized animals (42 ± 10.2, n = 8 and 47.8 ± 11.5 n = 11, p < 0.05 respectively for dose).

## Discussion

Inflammatory cell migration to the lung has a number of similarities with tumour cell metastasis, for example, secreting heparanase to degrade ECM structure. The results demonstrated that heparanase plays a greater role in regulating the pulmonary cell recruitment of eosinophils following an allergic, compared with neutrophils following a non-allergic, stimulus.

A previous study has reported that the constitutive cell population within the airways of mice deficient in heparanase was similar to the number of cells in naïve mice [23], an observation that was also observed in our study. It has been suggested that the deletion of the heparanase gene may lead to a compensatory increase in MMP’s primarily MMP-2, MMP-14 and MMP-9, which are known to degrade the ECM [24], although this is not a consistent finding [17]. Whether compensation by MMPs in the lung could explain why mice deficient in heparanase were able to recruit neutrophils in response to zymosan or LPS remains to be established.

We also used a pharmacological approach to inhibit heparanase activity in WT mice using the heparanase inhibitor, Muparfostat derived from yeast. This phosphosulfomannan has demonstrated efficacy against several cancers in phase I and II clinical trials, either administered alone or in combination with other therapy [25,26]. Muparfostat inhibits heparanase with an IC50 of 7.9 nM [27] and has previously been shown to suppress experimental lung metastases in a B16 melanoma model [28]. However, Muparfostat, in a dose range similar to those reported in this melanoma model did not significantly reduce the number of neutrophils infiltrating to the lung in response to zymosan or LPS in our study.

The current findings do not support the notion that heparanase is important in the acute innate inflammatory response in response to airway injury, although we have recently reported that recombinant heparanase is able to promote neutrophil recruitment into tissues [29]. It has also been reported that heparanase inhibition prevents sepsis-induced mortality and acute lung injury (ALI) in mice [16]. This apparent discrepancy may reflect pathophysiologic differences between direct (i.e. intranasal) and indirect (i.e. systemic-induced) lung injury, as suggested by previously-observed differences in patients with direct (pneumonia) and indirect (sepsis, pancreatitis) lung injury [16,30]. Alternatively, heparanase might be important during the early phase of the inflammatory response, as leukocyte attachment was measured 6 h after acute lung injury [16] whilst in our studies we measured leukocyte recruitment at a 24 h time point.

It has recently been shown that heparanase is involved in the chronic inflammatory response in ulcerative colitis [31] and chronic inflammation is known to have a profound effect on tumour progression [32]. Consequently, heparanase is a potential target for therapeutic intervention in chronic inflammatory disease states. We investigated the expression of heparanase in subjects with different severities of COPD and found an association of heparanase expression in lung tissue and COPD severity. This would be consistent with evidence of increased expression of this enzyme in other chronic inflammatory conditions including proteinuric disease [33], chronic haemodialysis [34], rheumatoid arthritis [13], Crohn’s disease and ulcerative colitis but not in infection colitis [12]. We cannot draw any conclusion concerning cause and effect, but our data highlight a further avenue for research regarding the role of heparanase in COPD.

Whilst mice deficient in heparanase have been shown to have no detectable difference in response to a DTH reaction [24], although there was a reduction in the number of monocytes recruited to the inflammatory site [15], a pharmacological approach using the *in vivo* administration of anti-heparanase siRNA or an inhibitor of heparanase enzymatic activity effectively abolished the DTH inflammatory response in the skin of mice [14]. Hence, we investigated the role of heparanase in allergic inflammation in the lung using an allergic model of murine lung inflammation which is characterized by a Th2 phenotype [35]. Mice sensitized and then exposed to aerosolized OVA demonstrated a significant increase in inflammatory cell influx, predominantly composed of eosinophils which was accompanied with changes in pulmonary lung mechanics, as evident by an increase in airways responsiveness to methacholine. We have demonstrated significantly lower number of eosinophils in the BAL fluid of Hpa^-/-^ mice sensitized and then subsequently challenged with ovalbumin. This is consistent with a report demonstrating that the ablation of heparanase attenuated eosinophil recruitment and Th2 cytokine response in house dust mite immunized mice [17]. This effect of heparanase is further demonstrated following pharmacological treatment of mice with a heparanase inhibitor. Moreover, we for the first time have demonstrated bronchial hyperresponsiveness (BHR) was suppressed following pharmacological inhibition of heparanase. To increase the generality of our findings we used two different strains of mice for studies on the role of heparanase in pulmonary allergic inflammation. Strain dependent differences in bronchial hyperresponsiveness following antigen challenge are well known [36] and the BALBc strain is more sensitive than C57Bl/6 strain for measuring physiological changes in respiratory lung mechanics.

There are a number of possible mechanisms to explain the decrease in eosinophil recruitment to the lung and the reduction of BHR following inhibition of heparanase. We and others have demonstrated that platelets are central to the recruitment of eosinophils in allergic inflammation [37–39]. In addition, activated, but not resting platelets were also necessary for the development of leukocyte complexes and the subsequent inflammatory response [40]. Platelet secreted heparanase [41] affects various signal transduction pathways following uptake into cells [42]. Other cell types involved in the allergic inflammatory response in the airways including dendritic cells [17,43], T cells [44–50] and vascular endothelium [16,50,51] also express heparanase to potentially regulate the migration of leukocytes to sites of inflammation. Selective ablation of heparanase in individual cell types would be required to investigate the role of heparanase expressing cells in the allergic inflammatory response and is beyond the scope of this study.

## Conclusions

We have demonstrated a role for heparanase in acute allergic inflammation, in the lung and our findings highlight a potential role for this enzyme in cell recruitment to the airways and that targeting heparanase may be beneficial in the treatment of allergic pulmonary diseases.
